# Obstructive colonic FGESF associated with feline infectious peritonitis in a cat: a case report

**DOI:** 10.1186/s12917-026-05287-0

**Published:** 2026-01-23

**Authors:** Masoud Navvabi, Reza Samaei, Vahid Fathipour, Mehras Mazandarani, Fatemeh Askarzadeh, Mohammad Nasrollahzadeh Masouleh, Arman Abdous

**Affiliations:** 1 Goldenfox Pet Clinic, Tehran, Iran; 2 Proba Veterinary Laboratory, Tehran, Iran; 3 Dr. Masouleh Veterinary Imaging Center, Tehran, Iran; 4 Department of Clinical Sciences,Ka.C., Islamic Azad University, Alborz, Iran; 5 Department of Clinical Sciences,SR.C., Islamic Azad University, Tehran, Iran; 6 School of Public Health, Tehran University of Medical Sciences, Tehran, Iran

**Keywords:** Feline infectious peritonitis, Feline coronavirus, Feline gastrointestinal eosinophilic sclerosing fibroplasia, GS-441524, Case report

## Abstract

**Background:**

Feline infectious peritonitis (FIP) is a severe immune-mediated disease that develops in a small proportion of cats infected with feline coronavirus (FCoV). Clinical manifestations are variable and most commonly involve systemic inflammatory disease with or without effusion. Gastrointestinal signs, including vomiting or diarrhea, may be observed; however, focal intestinal lesions leading to mechanical obstruction are rare. Histopathologic changes resembling feline gastrointestinal eosinophilic sclerosing fibroplasia (FGESF) have only rarely been reported in association with FIP.

**Case presentation:**

A 6-month-old Scottish Fold cat was evaluated for vomiting, constipation, weight loss, and jaundice. Abdominal radiography, ultrasonography, and contrast-enhanced computed tomography identified an approximately 80 mm segment of marked transmural thickening of the distal descending colon resulting in mechanical obstruction. Surgical resection was performed. Histopathologic examination of the excised segment revealed a dense fibroinflammatory lesion characterized by marked eosinophilic infiltration, reactive spindle-shaped fibroblasts, and prominent collagen trabeculae, consistent with an FGESF-like process. Immunohistochemistry demonstrated intracytoplasmic feline coronavirus antigen within macrophages in the resected tissue. Feline coronavirus RNA was also detected in peripheral blood by reverse-transcription polymerase chain reaction.

**Treatment and outcome:**

The affected colonic segment was resected with end-to-end anastomosis. Postoperative management included supportive care, antimicrobial therapy, corticosteroids, nutritional support via an esophagostomy tube, and antiviral treatment with GS-441,524. Antiviral therapy was initiated by subcutaneous injection and subsequently continued orally for a total duration of 12 weeks. Progressive clinical improvement was observed, including resolution of gastrointestinal signs and jaundice, weight gain, and improvement in clinicopathologic abnormalities during follow-up.

**Conclusions:**

This case describes an uncommon presentation of non-effusive FIP associated with a colonic FGESF-like fibroinflammatory lesion causing mechanical obstruction. FIP should be considered among differential diagnoses for eosinophilic intestinal masses in young cats, even in the absence of effusion. Integration of histopathology with molecular diagnostic techniques, including lesion-based immunohistochemistry, may support diagnostic evaluation in atypical cases. Early recognition and etiology-directed management may contribute to favorable short-term outcomes.

## Background

### Introduction to feline infectious peritonitis (FIP)

Feline infectious peritonitis (FIP) is an immune-mediated disease that develops in a small proportion of cats infected with feline coronavirus (FCoV). Although FCoV infection is common and often subclinical, only a minority of infected cats progress to FIP, reflecting complex host–virus interactions rather than a single obligatory viral mutation event [1]. Clinically, FIP is categorized into effusive and non-effusive forms, with overlap between presentations. The effusive form is characterized by immune-mediated vasculitis and protein-rich effusions, whereas the non-effusive form is associated with granulomatous or pyogranulomatous lesions affecting multiple organs [2]. Definitive diagnosis relies on integration of clinical findings with histopathology, with immunohistochemical detection of FCoV antigen within macrophages considered the diagnostic gold standard. Reverse-transcription polymerase chain reaction is supportive and must be interpreted in clinical context [3]. Gastrointestinal involvement in FIP is uncommon and typically limited to nonspecific clinical signs. Rarely, localized transmural intestinal lesions have been reported that may appear mass-like or obstructive and mimic feline gastrointestinal eosinophilic sclerosing fibroplasia (FGESF) on imaging, creating diagnostic uncertainty, particularly in young cats, as first described by Harvey et al. (1996) and subsequently reported in later series [4–6].

### Unusual presentations: gastrointestinal involvement

When gastrointestinal involvement occurs in feline infectious peritonitis (FIP), it most often manifests as nonspecific clinical signs such as vomiting, diarrhea, or anorexia and is generally attributed to systemic inflammation rather than primary intestinal disease [4, 7, 8]. Primary localized gastrointestinal lesions associated with FIP are infrequently reported [9]. Described lesions typically involve mural or serosal inflammatory or granulomatous changes with concurrent mesenteric lymphadenopathy rather than discrete intramural mass-forming lesions. However, rare cases document focal transmural granulomatous inflammation with fibrosis, resulting in localized bowel wall thickening that may closely resemble feline gastrointestinal eosinophilic sclerosing fibroplasia (FGESF) on imaging and histopathology, thereby posing diagnostic challenges [5, 9]. Because FGESF is defined by dense eosinophilic infiltration, reactive fibroblasts, and prominent collagen trabeculae, and is thought to arise from multifactorial immune dysregulation or infectious triggers [10, 11], histologic overlap between these entities may lead to diagnostic uncertainty. This highlights the importance of including feline infectious peritonitis (FIP) in differential diagnoses and confirming etiology through lesion-based immunohistochemistry and targeted molecular testing when appropriate [9].

### Literature review and case uniqueness

Reported gastrointestinal lesions in feline infectious peritonitis most commonly reflect macrophage-driven inflammatory or granulomatous processes involving serosal surfaces and mesenteric lymph nodes, whereas intramural mass-forming lesions represent atypical and only sporadically described manifestations of the disease [4, 5, 9]. In contrast, feline gastrointestinal eosinophilic sclerosing fibroplasia (FGESF) is an uncommon fibroinflammatory condition characterized by transmural fibrosis, dense eosinophilic infiltration, and fibroblastic proliferation, and has been associated with immune dysregulation and diverse potential triggers, including bacterial infections rather than feline coronavirus [10–12].

Within this context, the present case describes a young cat with confirmed feline coronavirus infection and a colonic mass exhibiting histologic features characteristic of FGESF. In the absence of lesion-based immunohistochemistry or molecular testing, such a lesion could reasonably be misclassified as idiopathic FGESF or neoplasia. This case therefore emphasizes a critical diagnostic overlap between FIP-associated gastrointestinal fibroinflammatory lesions and FGESF-like pathology and underscores the importance of comprehensive diagnostic evaluation, including pathogen-specific testing, in young cats presenting with eosinophilic intestinal masses or obstructive gastrointestinal disease.

### Case presentation

#### Patient information

**Fig. 1:**
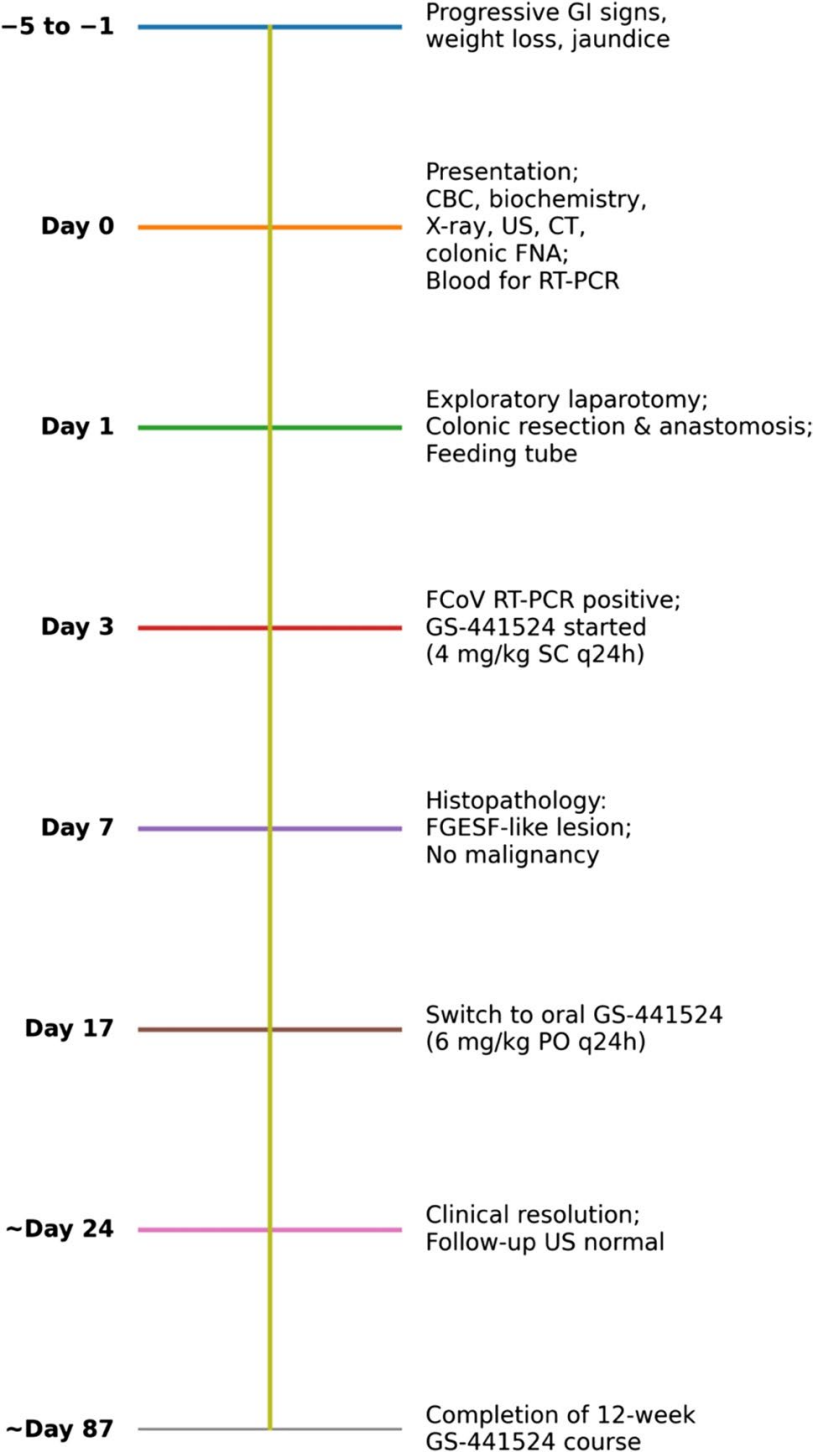
Clinical timeline of diagnostics, surgical intervention, antiviral therapy, and follow-up in a cat with non-effusive feline infectious peritonitis presenting as an FGESF-like obstructive colonic lesion

A 6-month-old intact female Scottish Fold cat was referred for evaluation of a 5-day history of vomiting, progressive inappetence, and constipation. The owner reported recent weight loss, lethargy, and reduced interaction with normal activities. The cat was housed exclusively indoors, had no known exposure to other animals, and was current on vaccinations and routine deworming. There was no known history of trauma, foreign body ingestion, toxin exposure, prior medical illness, or previous medical or surgical intervention. During the 48 h preceding presentation, the cat developed repeated unsuccessful attempts to defecate and recurrent vomiting of partially digested food and gastric fluid. Worsening lethargy and anorexia prompted urgent veterinary evaluation. A chronological summary of key clinical events, diagnostic procedures, therapeutic interventions, and outcomes is provided in Fig. 1.

#### Clinical findings

On physical examination, the cat was quiet and depressed, anorexic, and moderately dehydrated, estimated at 6–8%, with evidence of weight loss. Abdominal palpation revealed generalized distension and marked discomfort, consistent with a gastrointestinal obstructive process. No discrete mass or foreign body was palpable. Vital parameters included a rectal temperature of 39.5 °C and mild tachycardia. The mucous membranes were tacky, with a mildly prolonged capillary refill time. These findings were consistent with dehydration and early physiological compromise, prompting immediate diagnostic evaluation, including laboratory testing and diagnostic imaging.

### Investigations

#### Hematology and serum biochemistry

On admission, a complete blood count revealed mild anemia with a hematocrit of 32%, neutrophilia with a left shift at 10,500/µL, lymphopenia at 800/µL, and eosinophilia at 2,000/µL. These hematologic abnormalities were nonspecific and compatible with inflammatory, infectious, or immune-mediated disease processes. Serum biochemistry showed increased alanine aminotransferase at 180 U/L and aspartate aminotransferase at 120 U/L, consistent with hepatocellular injury. Hypoalbuminemia of 2.0 g/dL was also identified and considered potentially multifactorial, including contributions from systemic inflammation, gastrointestinal protein loss, or reduced hepatic synthesis. Blood urea nitrogen and creatinine concentrations were within reference intervals, indicating preserved renal function (Table 1). Overall, the hematologic and biochemical findings supported the presence of systemic illness but were not diagnostic of a specific underlying disease. Given the progressive gastrointestinal signs, abdominal pain, and laboratory abnormalities, further diagnostic evaluation was pursued.

**Table 1:** Hematology and serum biochemistry parameters at presentation and during Follow-up

Parameter	Day 0	Week 1	Week 2	Week 3	Week 6	Week 9	Week 12	Reference range
Hematocrit (%)	32	35	36	38	38	40	41	35–45
Neutrophils (/µL)	10,500	9,200	8,600	7,900	7,200	6,700	6,500	3,000–12,500
Lymphocytes (/µL)	800	1,100	1,300	1,500	1,900	2,000	1,900	1,500–7,000
Eosinophils (/µL)	2,000	1,350	1,120	980	900	750	680	0–1,500
ALT (U/L)	183	135	121	95	79	68	60	10–100
AST (U/L)	121	83	65	48	39	32	28	10–50
Albumin (g/dL)	2.0	2.1	2.4	2.5	2.7	2.9	3.0	2.5–4.0
Globulin (g/dL)	4.8	4.5	4.2	4.0	3.7	3.7	3.8	2.5–4.5
Albumin: globulin ratio	0.42	0.47	0.57	0.63	0.73	0.78	0.79	> 0.6
Total bilirubin (mg/dL)	2.1	1.2	1.0	0.8	0.5	0.2	0.2	< 0.4
BUN (mg/dL)	18	19	21	22	21	24	27	16–36
Creatinine (mg/dL)	1.3	1.1	1.0	1.1	1.4	1.1	1.2	0.6–1.8

GS-441,524 treatment was initiated on Day 3 following presentation

### Diagnostic imaging

#### Abdominal radiography

**Fig. 2:**
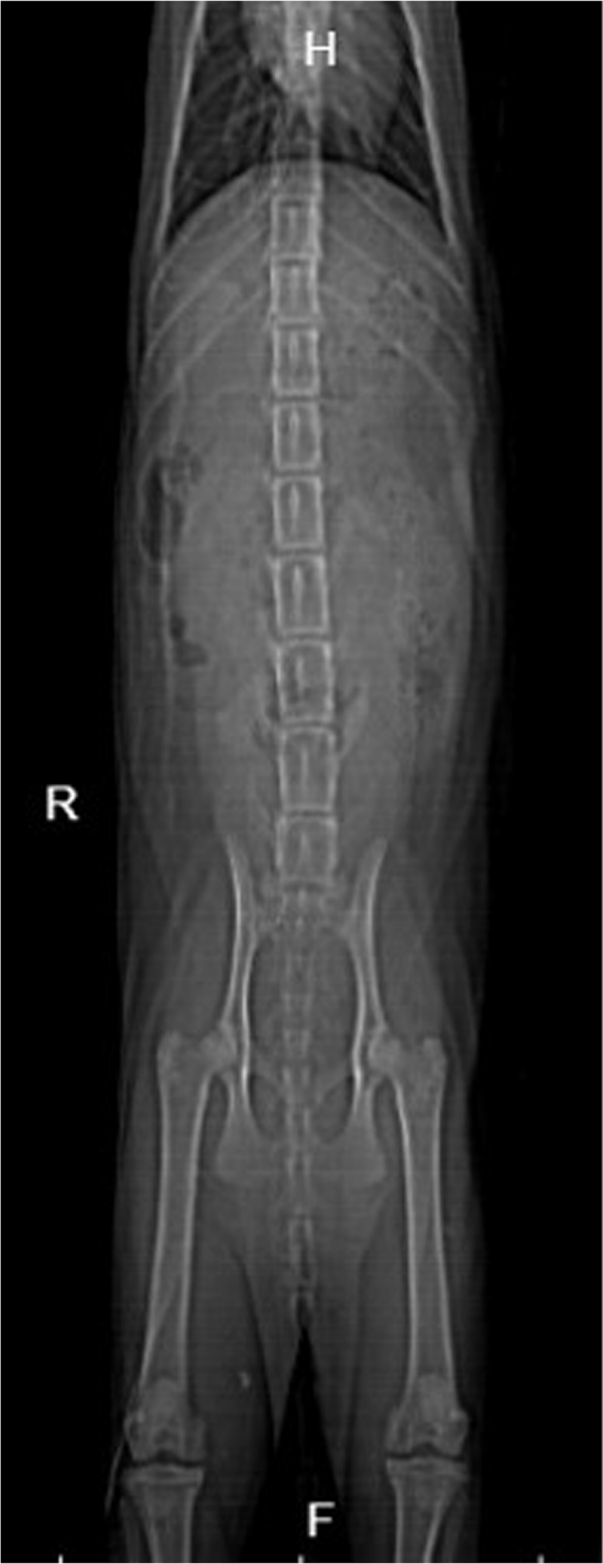
Ventrodorsal abdominal radiograph. Reduced abdominal serosal detail and paucity of colonic gas, findings considered nonspecific in a young cat with limited intra-abdominal fat. No radiopaque foreign material, abnormal visceral displacement, or definitive evidence of mechanical obstruction is identified

Right lateral and ventrodorsal abdominal radiographs demonstrated alteration of the normal colonic gas pattern and reduced abdominal serosal detail. These findings were considered nonspecific and potentially attributable to the patient’s young age, limited intraperitoneal fat, or the presence of a small volume of free fluid. No radiopaque foreign material, abnormal visceral displacement, or discrete intraluminal obstruction was identified (Fig. 2).

#### Abdominal ultrasonography

**Fig. 3:**
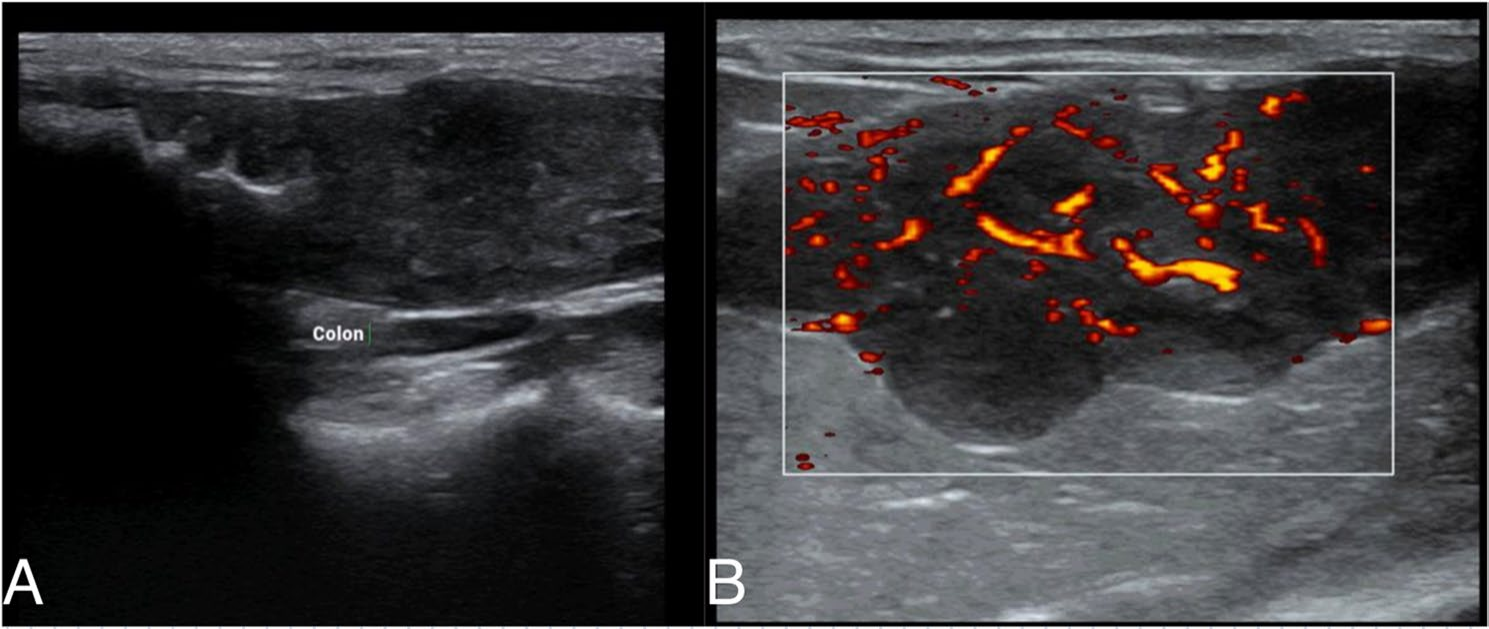
Abdominal ultrasonography of the distal descending colon. **a** Greyscale image demonstrating circumferential hypoechoic mural thickening (≈ 5–7 mm) with complete loss of normal wall layering and luminal narrowing. **b** Color Doppler image showing increased intralesional and peripheral vascularity (arrows), consistent with an active inflammatory or infiltrative mural process

Abdominal ultrasonography revealed marked circumferential thickening of the distal descending colon with complete loss of normal wall layering and increased intralesional and peripheral vascularity. The affected colonic wall was hypoechoic and irregular, measuring approximately 5–7 mm in thickness, compared with a reference value of ≤ 1.5 mm, indicating severe mural involvement. The medial iliac lymph nodes were enlarged. Based on established sonographic criteria [13], these findings raised concern for an infiltrative or neoplastic process, although severe inflammatory disease could not be excluded. No free abdominal fluid was detected, and the remaining abdominal organs appeared within normal limits (Fig. 3).

#### Computed tomography (CT)

**Fig. 4:**
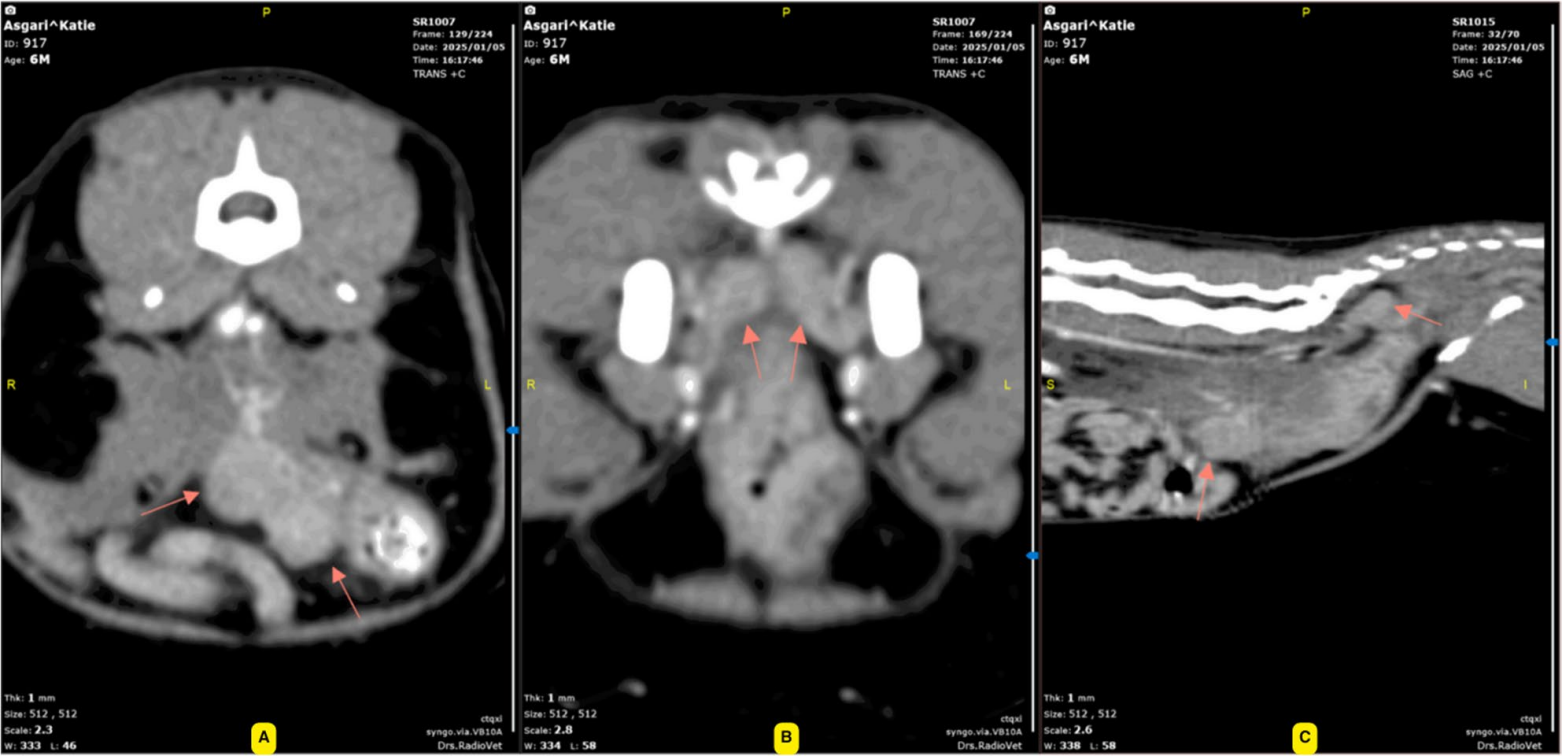
Contrast-enhanced computed tomography (CT) of the distal descending colon extending toward the rectum. **A** Transverse post-contrast image demonstrating marked circumferential transmural thickening of the distal descending colon with severe luminal narrowing (arrows), resulting in mechanical obstruction. The affected colonic wall shows heterogeneous contrast enhancement. **B** Dorsal reformatted post-contrast image illustrating the longitudinal extent (approximately 80 mm) of the colonic lesion (arrows) with relatively well-defined proximal and distal margins and associated proximal colonic dilation. **C** Sagittal reformatted post-contrast image showing heterogeneous enhancement of the thickened colonic wall (arrows) and mild haziness of the adjacent mesocolic fat, consistent with an active inflammatory or infiltrative process.Enlarged medial iliac and sacral lymph nodes with strong contrast enhancement were also identified on additional slices, while no ascites or evidence of distant metastatic disease was detected

Pre- and post-contrast abdominal computed tomography confirmed an approximately 80 mm segment of transmural thickening involving the distal colon and rectum, resulting in marked luminal narrowing and mechanical obstruction proximal to the lesion. The affected segment showed heterogeneous attenuation on pre-contrast images and marked contrast enhancement following intravenous contrast administration, measuring approximately 50–110 Hounsfield units. The surrounding mesocolic fat appeared mildly hazy, and the medial iliac and sacral lymph nodes were enlarged with strong contrast enhancement. No ascites or evidence of distant involvement of the liver, spleen, or visualized portions of the lungs was identified (Fig. 4).

### Cytologic evaluation

Ultrasound-guided fine-needle aspiration of the transmural colonic lesion yielded moderately cellular samples dominated by a dense eosinophilic infiltrate. Eosinophils were admixed with reactive fibroblasts and a fibrillar extracellular matrix consistent with fibrotic stroma. Occasional spindle-shaped mesenchymal cells were present, without significant cytologic atypia or increased mitotic activity. No epithelial cell clusters, monomorphic lymphoid populations, or cytologic features suggestive of carcinoma, lymphoma, or mast cell tumor were identified. Although cytologic evaluation cannot definitively exclude neoplasia, the findings were considered more consistent with a non-neoplastic fibroinflammatory process. Overall, the cytologic features supported an eosinophilic fibrosing inflammatory lesion consistent with feline gastrointestinal eosinophilic sclerosing fibroplasia (FGESF) and other eosinophilic gastrointestinal disorders. In combination with the imaging findings, these results supported the decision to pursue exploratory laparotomy and surgical biopsy for definitive diagnosis.

### Diagnostic challenges

Definitive diagnosis was complicated by the absence of abdominal effusion and the presence of a focal eosinophilic fibroinflammatory colonic mass with imaging and cytologic features overlapping those of idiopathic FGESF and intestinal neoplasia. These factors limited the diagnostic specificity of imaging, cytology, and blood-based testing alone and necessitated integration of histopathology with lesion-based pathogen detection.

### Immunohistochemistry for feline coronavirus

Immunohistochemical staining for feline coronavirus (FCoV) antigen was performed on formalin-fixed, paraffin-embedded sections of the resected colonic lesion at Proba Veterinary Laboratory in Tehran, Iran. Tissue sections were deparaffinized and rehydrated, followed by heat-induced antigen retrieval using citrate buffer at pH 6.0. Sections were incubated with a mouse monoclonal antibody directed against the feline coronavirus nucleocapsid protein (clone FIPV3-70; Custom Monoclonals International, USA). Antibody binding was visualized using a polymer-based detection system with 3,3′-diaminobenzidine chromogen and hematoxylin counterstaining. Appropriate positive and negative controls were included. Focal intracytoplasmic immunoreactivity was identified within macrophages in the lesion.

### RT-PCR for feline coronavirus

Reverse-transcription polymerase chain reaction testing for feline coronavirus RNA was performed on an EDTA-anticoagulated whole-blood sample submitted to Proba Veterinary Laboratory in Tehran, Iran. Samples were collected under aseptic conditions and stored at − 80 °C until analysis. Total RNA was extracted using a commercial viral RNA extraction kit (AddBio, South Korea) according to the manufacturer’s instructions. Detection of FCoV RNA was conducted using a one-step real-time RT-PCR assay with primers and a probe targeting FCoV-specific sequences (forward primer: GCATTTACTCTAATAGATGACC; reverse primer: CTTCGTCGGGAATATATGCC; probe: FAM-ATATTGTTCCCAATAGCATTGCTAAACG-BHQ1). Amplification and fluorescence detection were performed on an ABI StepOnePlus Real-Time PCR System (Applied Biosystems, USA). Each PCR run included a positive control and a no-template control. Results were interpreted qualitatively, and samples demonstrating exponential amplification with a cycle threshold value within the accepted assay range were considered positive. The RT-PCR result was positive, indicating systemic FCoV infection. In accordance with current diagnostic guidelines, blood-based RT-PCR was interpreted as supportive but not independently diagnostic for feline infectious peritonitis, whereas lesion-based immunohistochemistry demonstrating intracytoplasmic FCoV antigen within macrophages provided confirmatory diagnostic evidence in this case.

### Prognosis

At the time of diagnosis, the prognosis was considered guarded because of suspected non-effusive feline infectious peritonitis, the presence of a severe focal colonic lesion causing mechanical obstruction, and the requirement for surgical intervention. However, the absence of neurologic or ocular involvement and the ability to achieve complete surgical resection of the obstructive lesion were considered potentially favorable prognostic factors, provided that effective antiviral therapy could be initiated promptly.

#### Treatment

The cat was admitted in a clinically unstable condition characterized by progressive gastrointestinal obstruction, weight loss, anorexia, and jaundice. Given the deterioration in clinical status and the presence of a severe mural colonic lesion identified on imaging and cytology, exploratory laparotomy was selected as both a diagnostic and therapeutic intervention.

**Fig. 5:**
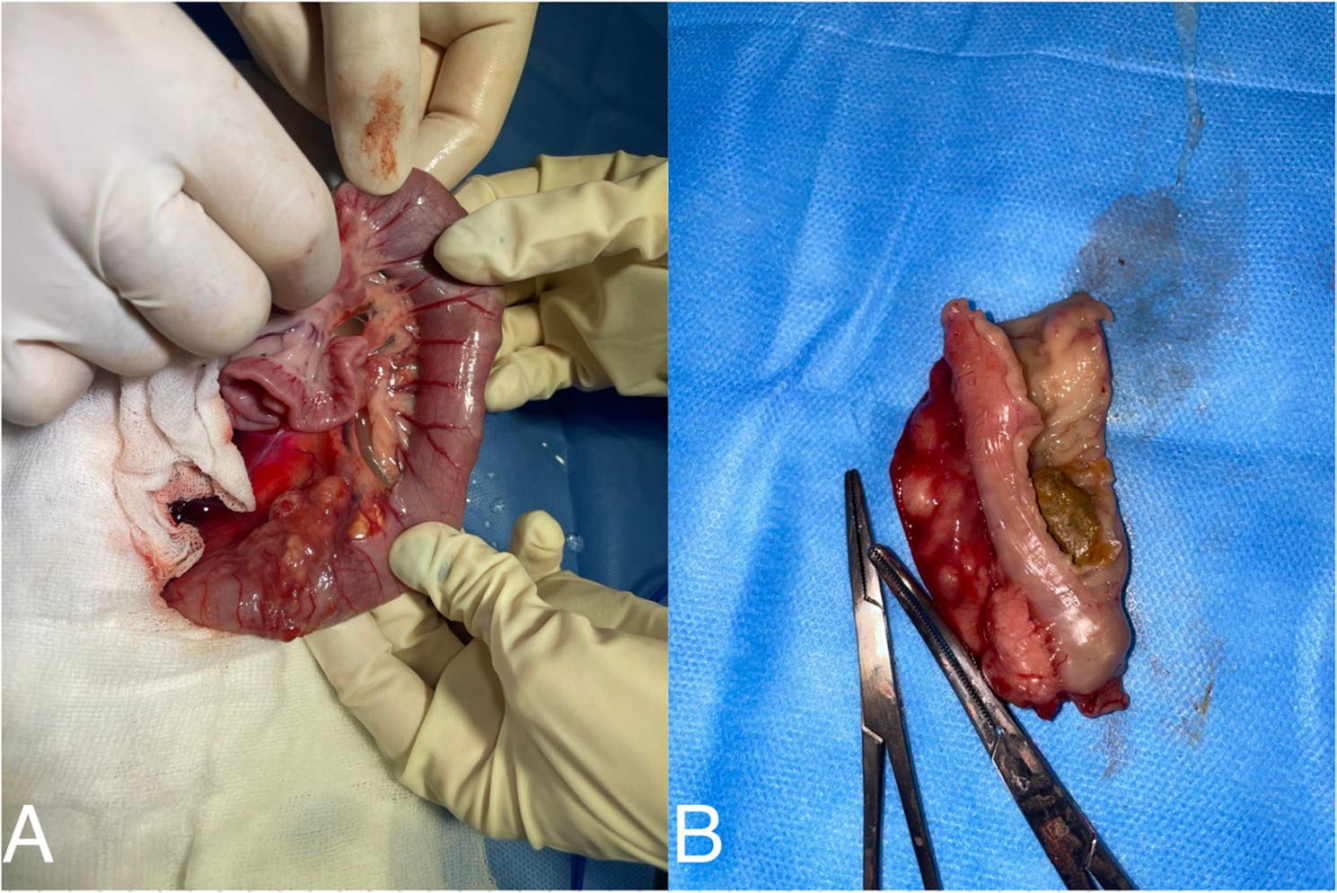
Gross appearance of the distal colon. **a** Resected segment showing circumferential mural thickening with severe luminal narrowing and inspissated fecal material proximal to the stricture. **b** Opened specimen demonstrating irregular, firm fibroinflammatory tissue expanding the colonic wall

At surgery, a firm, markedly thickened, fibrotic segment of the distal descending colon was identified, resulting in severe luminal narrowing. The affected intestinal segment was resected, and intestinal continuity was restored using an end-to-end anastomosis (Fig. 5). No gross evidence of intestinal perforation, generalized peritonitis, or metastatic disease was observed. The excised tissue was submitted for histopathologic examination.

Postoperative management consisted of intensive supportive care. Intravenous crystalloid fluids, specifically lactated Ringer’s solution at approximately 60 mL/kg/day, were administered during the first 48 h and subsequently adjusted based on hydration status, urine output, and clinical response. Broad-spectrum antimicrobial therapy was initiated to reduce the risk of bacterial translocation associated with severe colonic inflammation and intestinal surgery. This included cefazolin at 22 mg/kg intravenously every 12 h, metronidazole at 15 mg/kg intravenously every 12 h, and enrofloxacin at 5 mg/kg subcutaneously every 24 h for a total of seven days.

Anti-inflammatory therapy was initiated with dexamethasone at 0.1 mg/kg administered intramuscularly once daily for five days to manage a suspected immune-mediated inflammatory process. This decision was based on marked peripheral eosinophilia, severe transmural intestinal inflammation, and progressive clinical deterioration at a time when a definitive etiologic diagnosis had not yet been established. Corticosteroid therapy was selected over nonsteroidal anti-inflammatory drugs because of severe gastrointestinal disease, recent colonic resection with end-to-end anastomosis, and anorexia, as nonsteroidal anti-inflammatory drugs are associated with an increased risk of gastrointestinal ulceration and impaired mucosal healing under these conditions. Following histopathologic confirmation of an FGESF-like eosinophilic fibroinflammatory lesion, the short-term use of corticosteroids was considered appropriate and consistent with published management strategies for eosinophil-driven intestinal inflammation [14, 15]. By one week postoperatively, peripheral eosinophil counts had decreased substantially on complete blood count evaluation, and no further corticosteroid therapy was administered. The patient was monitored daily with physical examinations, serial hematologic and biochemical assessments, and careful evaluation of gastrointestinal function and surgical site healing.

After detection of feline coronavirus RNA in peripheral blood by reverse-transcription polymerase chain reaction, antiviral therapy with GS-441,524 was initiated at a dose of 4.0 mg/kg administered subcutaneously once daily for 14 days. This initial parenteral phase was chosen because of postoperative anorexia and concern for unreliable oral absorption. Following clinical stabilization and improvement in voluntary food intake, treatment was continued with oral GS-441,524 at a dose of 6.0 mg/kg once daily to complete a total treatment duration of 12 weeks. The dosing regimen was based on published experimental and clinical studies demonstrating the efficacy of daily GS-441,524 administration at approximately 4.0 mg/kg in cats with naturally occurring feline infectious peritonitis without neurologic or ocular involvement [9, 16, 17]. Although blood-based RT-PCR results were interpreted as supportive rather than independently diagnostic [3, 18], lesion-based immunohistochemistry demonstrating intracytoplasmic feline coronavirus antigen within macrophages provided confirmatory diagnostic evidence in this case.

Nutritional support was provided via an esophagostomy feeding tube placed at the time of surgery. A high-protein, calorie-dense recovery diet (Royal Canin Recovery) was administered at approximately 50 mL/kg/day divided into three meals. Over the subsequent two weeks, voluntary oral intake gradually improved, allowing transition to a digestible canned diet and subsequently to a maintenance diet.

During the postoperative and treatment period, gastrointestinal function progressively improved, with resolution of vomiting and constipation and gradual weight gain. Follow-up imaging was consistent with relief of mechanical obstruction, and serial laboratory testing demonstrated improvement in previously identified abnormalities throughout the 12-week follow-up period (Table 1). Although the long-term prognosis for non-effusive feline infectious peritonitis remains guarded [7, 8, 19], the short-term clinical outcome in this case was favorable following combined surgical management and antiviral therapy.

### Follow-up and outcomes

Following surgical resection and postoperative management, including antiviral therapy with GS-441,524, the cat showed progressive clinical improvement. Vomiting resolved, normal defecation resumed within several days, and jaundice gradually diminished with continued supportive care, short-term corticosteroid therapy, antiviral treatment, and nutritional support. By the end of the first postoperative week, weight gain was evident and the cat successfully transitioned to voluntary oral feeding.

**Fig. 6:**
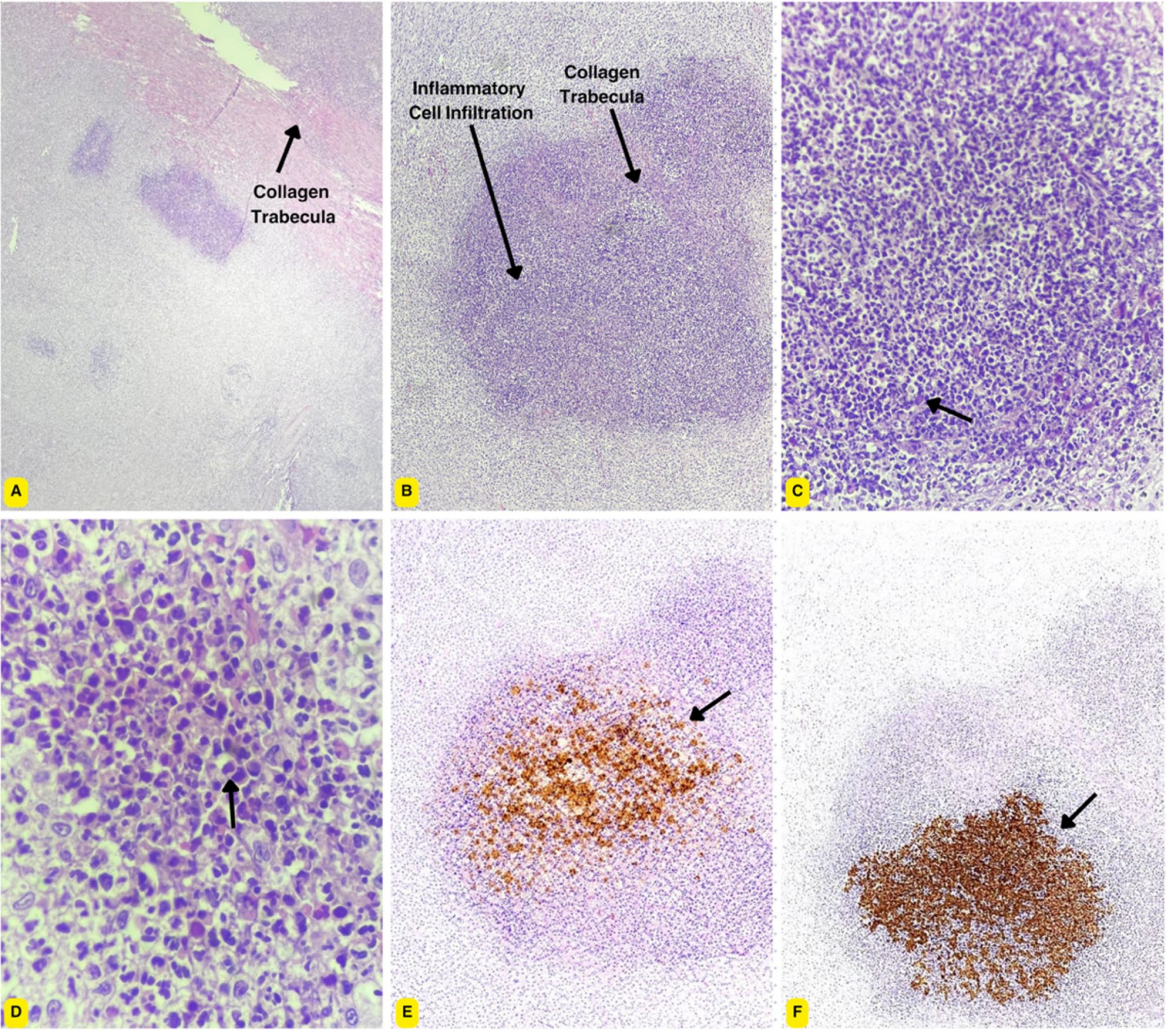
Histopathology and immunohistochemistry of the resected colonic lesion. **A** Low-power view (hematoxylin and eosin [H&E], 4× objective) showing a well-demarcated, nodular inflammatory lesion expanding the colonic wall with multifocal cellular aggregates and partial effacement of the normal architecture; a prominent collagenous trabecula within the lesion is indicated (arrow). **B** Intermediate magnification (H&E, 10× objective) demonstrating dense inflammatory cell infiltration arranged in nodular aggregates within a collagen-rich fibrous stromal background; collagenous trabeculae are highlighted (arrows). **C** Higher magnification (H&E, 40× objective) illustrating a dense mixed inflammatory infiltrate composed predominantly of eosinophils, admixed with macrophages, neutrophils, lymphocytes, and occasional stromal cells (arrow). **D** High-power view (H&E, 100× objective) highlighting numerous eosinophils infiltrating a collagen-rich stromal background, consistent with an eosinophil-rich fibroinflammatory lesion (arrow). **E** Immunohistochemistry highlighting eosinophils using an eosinophil granule protein marker, demonstrating abundant eosinophils distributed throughout the inflammatory lesion (DAB chromogen, hematoxylin counterstain; arrow). **F** Immunohistochemistry for feline coronavirus (FCoV) antigen demonstrating focal intracytoplasmic granular immunoreactivity within clusters of macrophages in the colonic lesion (DAB chromogen, hematoxylin counterstain; arrow)

Histopathologic evaluation of the resected colonic segment confirmed a dense fibroinflammatory process characterized by extensive eosinophilic infiltration, reactive fibroblasts, and prominent collagen trabeculae, morphologically consistent with feline gastrointestinal eosinophilic sclerosing fibroplasia (FGESF) [10, 14]. The inflammatory infiltrate also included spindle-shaped myofibroblasts, macrophages, neutrophils, and lymphocytes, with focal granulomatous inflammation and intralesional bacteria (Fig. 6). No histologic evidence of malignancy was identified, supporting a non-neoplastic inflammatory process.

Lesion-based immunohistochemistry demonstrated intracytoplasmic feline coronavirus antigen within macrophages, confirming feline infectious peritonitis in the appropriate clinicopathologic context [3, 4]. Together with compatible histopathologic findings, systemic clinical features, and supportive blood RT-PCR results, these findings substantiated the association between feline infectious peritonitis and the FGESF-like colonic lesion in this case.

The cat completed the full 12-week course of GS-441,524, consisting of an initial subcutaneous phase followed by oral administration, without observed adverse effects. Treatment adherence was assessed through owner reporting, clinical monitoring, and serial examinations. No injection-site reactions, gastrointestinal intolerance, or laboratory abnormalities attributable to antiviral therapy were detected, consistent with previously reported safety profiles [16, 17].

By week three, abdominal discomfort and clinical signs attributable to the obstructive colonic lesion had fully resolved. Serial hematologic and biochemical monitoring demonstrated progressive normalization or improvement of previously identified abnormalities. Hematocrit increased from 32% at admission to 36% by week one and 39% by week three. Neutrophil counts decreased from 10,500/µL to 7,800/µL, while lymphocyte counts increased from 800/µL to 1,600/µL. Eosinophil counts showed a progressive decline over the follow-up period, decreasing from 2,000/µL at admission to within the reference interval by the end of monitoring. Alanine aminotransferase decreased from approximately 180 U/L to 95 U/L, and aspartate aminotransferase from approximately 120 U/L to 48 U/L. Serum albumin increased from 2.0 g/dL to 2.7 g/dL, while blood urea nitrogen and creatinine concentrations remained within reference intervals throughout follow-up.

**Fig. 7:**
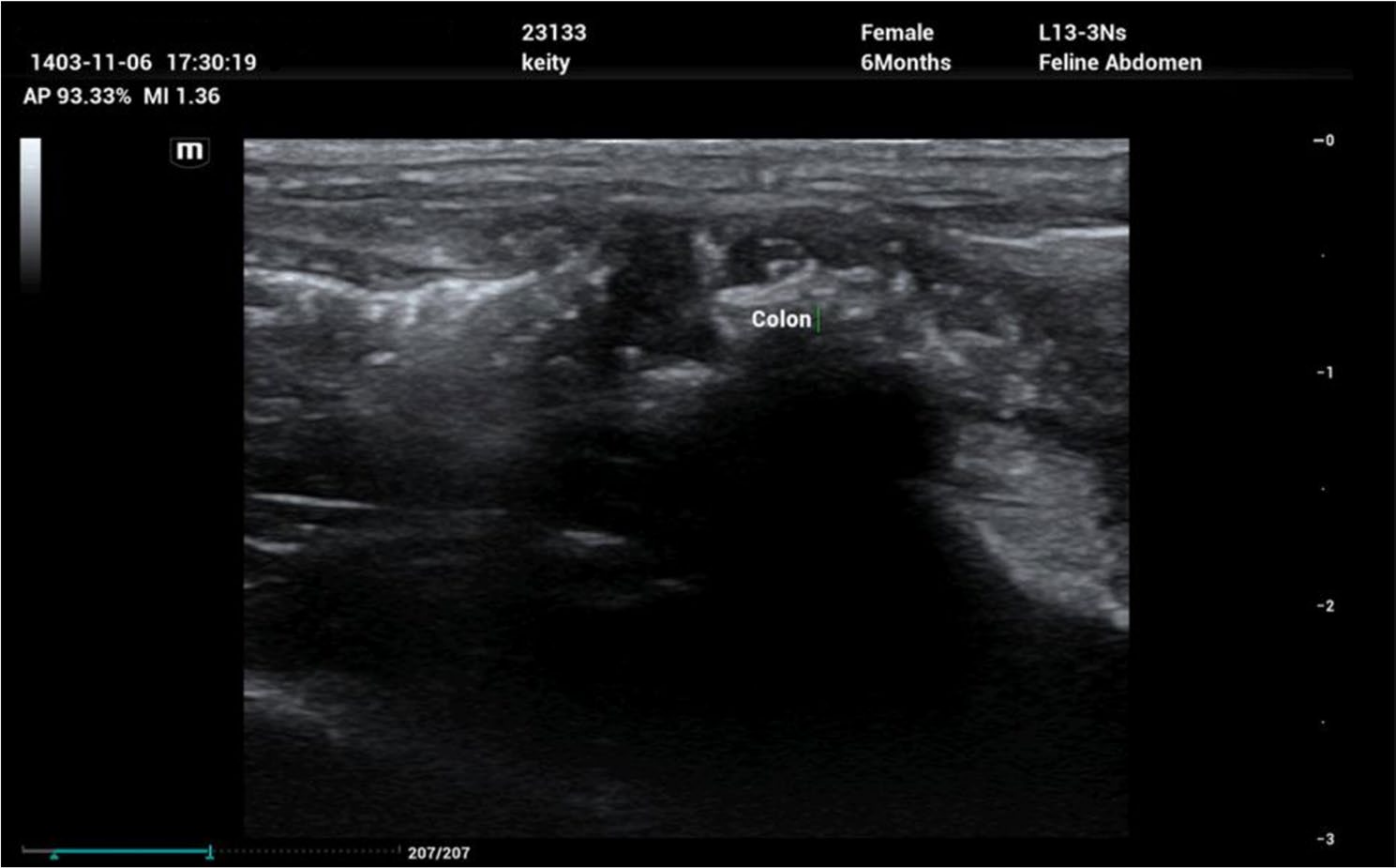
Postoperative abdominal ultrasonography.Longitudinal images of the distal colon obtained three weeks after surgery show restoration of normal wall thickness and layering, with no evidence of recurrent mural thickening or luminal obstruction

Repeat abdominal ultrasonography performed three weeks postoperatively demonstrated restoration of normal colonic wall thickness and layering, with no evidence of recurrent mural thickening, mass formation, or luminal obstruction, consistent with resolution of the previously documented obstruction (Fig. 7) [20]. Continued clinical improvement during follow-up supported a favorable short-term outcome. Although the long-term prognosis for cats with non-effusive feline infectious peritonitis remains guarded, particularly in atypical presentations [7, 8, 19], this case demonstrated a marked favorable short-term outcome following combined surgical management and antiviral therapy.

### Patient (Owner) perspective

The owner reported marked improvement in the cat’s appetite, activity level, and overall quality of life following surgery and initiation of antiviral therapy. Resolution of gastrointestinal signs and progressive weight gain were observed during follow-up, and the owner expressed satisfaction with the treatment outcome and postoperative recovery.

### Discussion and conclusions

## Discussion

Feline infectious peritonitis (FIP) is classically categorized into effusive and non-effusive forms and develops in a small proportion of cats infected with feline coronavirus (FCoV). Disease development reflects complex host–virus interactions and immune dysregulation, with viral genetic variation representing one of several proposed mechanisms rather than a single, universally defined mutation event [7, 8, 19]. Clinical manifestations are variable, and although fever is common, it is not obligatory, particularly in non-effusive or localized presentations [18]. While abdominal effusion, pyogranulomatous lesions, and systemic inflammation are well-recognized features of FIP, primary gastrointestinal involvement, especially mechanical obstruction caused by a focal mural lesion, has been reported only rarely [4, 15, 18]. Reviews by Addie et al. (2009) and Tasker et al. (2023) describe gastrointestinal signs in FIP, including vomiting and diarrhea, as typically mild and secondary to systemic disease rather than attributable to mass-forming lesions or mechanical obstruction [1, 4]. The present case expands the limited literature by describing non-effusive FIP presenting with colonic obstruction associated with an eosinophilic fibroinflammatory lesion exhibiting histologic features characteristic of feline gastrointestinal eosinophilic sclerosing fibroplasia (FGESF).

FGESF is generally considered idiopathic or associated with immune dysregulation, with proposed triggers including microbial agents, dietary hypersensitivity, parasitism, or foreign material [10, 11]. Systemic viral infections have not traditionally been implicated in its pathogenesis in published FGESF case series. In the absence of targeted etiologic testing, lesions such as the one described here could reasonably be misclassified as idiopathic FGESF or intestinal neoplasia, potentially delaying recognition of an underlying infectious cause. This underscores the importance of integrating imaging, cytology, histopathology, and pathogen-specific testing when evaluating eosinophilic intestinal masses in young cats.

A shared immunopathogenic mechanism may explain the overlap between FIP-associated lesions and FGESF-like pathology. In FGESF, profibrotic signaling pathways, including transforming growth factor-β1, have been implicated in eosinophil activation and fibroblast-driven collagen deposition [10]. Immune dysregulation associated with FCoV infection in FIP, characterized by macrophage activation and cytokine imbalance, may promote eosinophil recruitment and extracellular matrix remodeling within the intestinal wall. Activated eosinophils release mediators such as transforming growth factor-β1, matrix metalloproteinases, and eosinophil granule proteins, which can drive fibroplasia and mural thickening, mirroring mechanisms described in FGESF. Although causality cannot be established from a single case, coronavirus-associated immune dysregulation may initiate or mimic eosinophilic fibroplastic enteropathy in rare instances.

This case also highlights important diagnostic considerations. Imaging findings of marked transmural thickening with loss of wall layering and regional lymphadenopathy initially raised concern for neoplasia. However, cytologic identification of an eosinophil-rich fibroinflammatory process, followed by histopathologic demonstration of collagen trabeculae, redirected the diagnostic approach. Lesion-based immunohistochemistry identified intracytoplasmic feline coronavirus antigen within macrophages, providing confirmatory diagnostic evidence of FIP [3, 4], while blood-based RT-PCR was interpreted as supportive. Differential diagnoses for eosinophil-rich intestinal masses in cats should therefore include idiopathic FGESF, gastrointestinal lymphoma including T-cell variants, infectious eosinophilic granulomatous disease, and FIP-associated fibroinflammatory processes [20, 21].

The primary limitation of this report is its single-case design, which precludes generalization and prevents establishment of a causal relationship between feline infectious peritonitis and FGESF-like gastrointestinal lesions. In addition, long-term follow-up beyond completion of antiviral therapy was limited, and recurrence or delayed complications cannot be excluded. Nevertheless, the strength of this case lies in comprehensive clinicopathologic characterization, multimodal imaging, surgical confirmation, and integration of histopathology with pathogen-specific testing, allowing confident diagnosis of an atypical presentation of non-effusive feline infectious peritonitis.

### Clinical implications

This case has important clinical implications. Feline infectious peritonitis (FIP) should be included among the differential diagnoses for eosinophilic intestinal masses and obstructive gastrointestinal lesions in young cats, even in the absence of effusion. Accurate etiologic classification is therapeutically relevant, as demonstrated by resolution of obstruction, improvement in clinicopathologic abnormalities, and a favorable short-term outcome following combined surgical management, supportive care, and antiviral therapy with GS-441,524. Although the long-term prognosis for FIP remains guarded and conclusions cannot be generalized from a single case, early recognition of atypical presentations and etiology-directed intervention may improve short-term outcomes.

### Educational value

This case illustrates that pattern-based diagnostic assumptions, such as FIP being primarily effusive or FGESF being idiopathic, may not apply in atypical presentations. It emphasizes the need for a systematic diagnostic approach integrating clinical findings, imaging, cytology, histopathology, and pathogen-specific testing, including lesion-based immunohistochemistry, to resolve diagnostic uncertainty and avoid delayed or inappropriate management.

## Conclusion

This report expands the clinicopathologic spectrum of non-effusive FIP to include eosinophilic fibroinflammatory colonic obstruction with FGESF-like histology. It underscores the importance of considering FIP in young cats with eosinophilic gastrointestinal masses and demonstrates the diagnostic value of integrating histopathology with molecular and immunohistochemical testing. Increased awareness of this overlap may facilitate earlier diagnosis and improve short-term outcomes in similar atypical cases.

## Data Availability

The datasets used and/or analysed during the current study are available from the corresponding author on reasonable request **.**.

## Abbreviations

ALT: Alanine aminotransferase
AST: Aspartate aminotransferase
BUN: Blood urea nitrogen
CBC: Complete blood count
CT: Computed tomography
FCoV: Feline coronavirus
FIP: Feline infectious peritonitis
FGESF: Feline gastrointestinal eosinophilic sclerosing fibroplasia
FNA: Fine—needle aspiration
GI: Gastrointestinal
GS: 441524—Nucleoside analogue antiviral used in the treatment of FIP
HCT: Hematocrit
HU: Hounsfield units
IM: Intramuscular
IV: Intravenous
MMP: Matrix metalloproteinase
RT: PCR—Reverse—transcription polymerase chain reaction
SC: Subcutaneous
TGF: β1—Transforming growth factor beta 1
q12h/q24h: Every 12 h/every 24 h
